# Homo sapiens exhibit a distinct pattern of CNV genes regulation: an important role of miRNAs and SNPs in expression plasticity

**DOI:** 10.1038/srep12163

**Published:** 2015-07-16

**Authors:** Harsh Dweep, Nada Kubikova, Norbert Gretz, Konstantinos Voskarides, Kyriacos Felekkis

**Affiliations:** 1 Medical Research Center, University of Heidelberg, Mannheim, Germany; 2 Department of Life and Health Sciences, University of Nicosia, Nicosia Cyprus; 3 Molecular Medicine Research Center and Department of Biological Sciences, University of Cyprus, Nicosia, Cyprus; 4 St. George’s University of London Medical Program offered at the University of Nicosia Medical School, Nicosia, Cyprus

## Abstract

Gene expression regulation is a complex and highly organized process involving a variety of genomic factors. It is widely accepted that differences in gene expression can contribute to the phenotypic variability between species, and that their interpretation can aid in the understanding of the physiologic variability. CNVs and miRNAs are two major players in the regulation of expression plasticity and may be responsible for the unique phenotypic characteristics observed in different lineages. We have previously demonstrated that a close interaction between these two genomic elements may have contributed to the regulation of gene expression during evolution. This work presents the molecular interactions between CNV and non CNV genes with miRNAs and other genomic elements in eight different species. A comprehensive analysis of these interactions indicates a unique nature of human CNV genes regulation as compared to other species. By using genes with short 3′ UTR that abolish the “canonical” miRNA-dependent regulation, as a model, we demonstrate a distinct and tight regulation of human genes that might explain some of the unique features of human physiology. In addition, comparison of gene expression regulation between species indicated that there is a significant difference between humans and mice possibly questioning the effectiveness of the latest as experimental models of human diseases.

It is widely accepted that evolutionary phenotypic differences can be attributed to a great degree to gene expression variations. It has been suggested that major anatomical and physiological changes observed in organisms involve the evolution of complex gene expression networks^1^,^2^,^3^. A variety of genomic elements participate in the formation of these complexes but not yet completely understood gene expression regulatory networks may contribute to these evolutionary phenotypic differences.

Copy Number Variations (CNVs) refer to genomic regions with segmental duplications that have been recognized after systematic comparative genomic hybridizations using DNA of healthy human subjects. They are defined as DNA segments of 1 kb or larger in size more found at a significant frequency in the population^4^,^5^,^6^. CNVs are prevalent throughout the genome of humans and other species at both coding and non-coding regions and might include both genes and regulatory elements. MicroRNAs (miRNAs) constitute a class of short endogenous non-coding RNA molecules of 21–25 nucleotides (nt) in length, which function as negative regulators of gene expression at the post-transcriptional level in multicellular eukaryotes^4^. In addition to the mechanisms for gene regulation proposed, miRNAs can regulate a plethora of protein coding genes in humans and other species, primarily through binding to the 3′ UTR of the mRNA of target genes^4^.

Both miRNAs and CNVs are believed to contribute to expression plasticity and may be responsible for the unique phenotypic characteristics observed in the human lineage. In view of such a role, a comprehensive analysis of the interactions between miRNAs and CNVs in different species could provide an insight into the gene expression changes, which may have contributed to the appearance of new phenotypic characteristics in humans.

CNVs are known to exhibit genic bias. These genomic elements were shown to drive evolution and at the same time contribute to the pathogenesis of various human diseases. As a result, the regulation of expression and expression plasticity of genes lying in CNV regions could be of considerable interest in gaining an inside in the appearance of human-specific phenotypic characteristics. We have previously shown that miRNAs, evolutionary contribute to the regulation of CNV genes as this category of genes was found to harbor more miRNA binding sites and regulated by more miRNAs as compared to their non-CNV counterparts^7^. miRNA-CNV genes interactions were further explored to demonstrate the unique nature of the human lineage as compared to the other species. The nature of such interactions might explain some of the unique physiological attributes observed in *Homo sapiens*. We wanted to further explore the expression regulation of CNV genes and to compare the factors involved in this process. Through a comprehensive *in silico* analysis we explored the interrelations between CNV genes and other genomic factors such as miRNAs and SNPs. By using as a model, genes with short 3′UTR, which are expected to lose the “canonical” miRNA regulation, we were able to demonstrate a unique redistribution of miRNA binding sites and SNPs on CNV genes of human lineage. Human CNV genes appear to require overall a more tight and complex regulation, which is significantly different from any other species. In addition, our results provide an insight into important differences between humans and other species in regards to gene expression regulation that might have an impact on medicine and biology.

## Results

### Genes with short 3′ UTRs (less than 7 nt long), which are expected to abolish the “canonical” miRNA-dependent expression regulation

Regulation of gene expression is a complex and multifactorial process. Genes can be regulated simultaneously by a variety of genomic elements. Even miRNA-dependent regulation of gene expression follows different mechanistic processes. The major regulatory pathway for gene expression, however, involves the binding of miRNAs to target sites within the 3′ UTR region. In order to understand the importance of miRNA regulation of CNV and non-CNV genes in various species we used as a model, genes with short 3′ UTRs (less than 7 nts long) from eight different species [chicken (gga), dog (cfa), cow (bta), rat (rno), mouse (mmu), macaque (mml), chimpanzee (ptr), human (hsa)]. These genes are expected to lose the “canonical” miRNA-dependent regulation since miRNA will be unable to bind to that region.

Although, miRNAs are believed to regulate gene expression by targeting other genes regions, the major regulatory pathway involves the binding of miRNAs to target sites within the 3′ UTR region. Various studies have identified the presence of short 3′ UTR protein coding genes in the genome of various species including humans^8^,^9^,^10^,^11^. In general, it appears that the majority of 3′ UTR are relatively short. Liu *et al.* demonstrated that more than 50% of the 3′ UTR sequences examined in 22 different species have length between 100–300 bps^8^. Additional studies, have demonstrated that transcripts with shorter 3′ UTR in humans and zebrafish display higher level of expression and differential tissue expression attributing it to the loss of miRNA binding sites^10^,^11^. Despite these data, the link between loss of miRNA binding sites and gene expression has not been directly examined.

**Figure 1:**
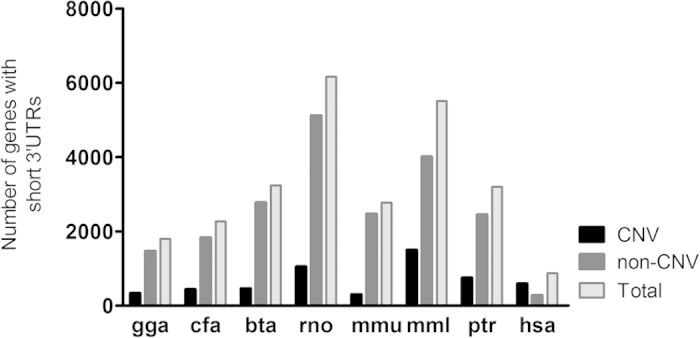
Number of genes with short 3′ UTR among 8 species. Comparison of the number of CNV versus non-CNV genes with short 3′ UTR in chicken (gga), dog (cfa), cow (bta), rat (rno), mouse (mmu), macaque (mml), chimpanzee (ptr), and human (hsa).

**Figure 2:**
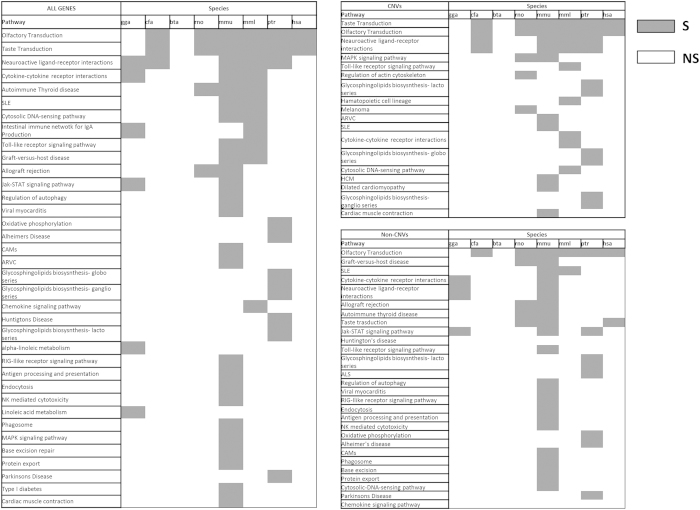
Significant Pathways on short 3′ UTR genes in the 8 different species: chicken (gga), dog (cfa), cow (bta), rat (rno), mouse (mmu), macaque (mml), chimpanzee (ptr), and human (hsa). Cells with dark and white background colors stand for significantly enriched and non-significant pathways, respectively.

We identified genes in both CNV and non-CNV regions in all of the eight species that have a 3′ UTR shorter than 7 nts (referred in text as “short 3′ UTR genes”). As depicted in Fig. 1, humans have fewer genes with short 3′ UTRs than all the other seven species, 869 compared to the next lowest 2,267 in dog. Of note, during the classification of these genes into CNV and non-CNV regions, we observed that the number of short 3′ UTR genes categorized under the non-CNV regions is higher than those categorized under CNV regions for all other species (excluding human), whereas, in case of human, an exact opposite trend is noted suggesting an alternative manner in the regulation of these genes in the human lineage. In order to identify any functional involvement, short 3′ UTR genes from all three categories (all genes, CNV genes and non-CNV genes) were interrogated against all known KEGG pathways. A total of 36 significantly enriched pathways were obtained for the all genes category (Fig. 2). Interestingly, two pathways appear to be significantly enriched with short 3′ UTR genes in the majority of species in all three categories. Olfactory- and Taste-transduction pathways were found to be significantly enriched for six (cfa, hsa, mml, mmu, ptr and rno) out of eight species. These pathways have been previously found to be associated with CNV regions and evolution demonstrating the genic bias exhibited in CNV regions and may reflect the diversity in senses in various organisms^12^,^13^,^14^.

As shown in Fig. 2, short 3′ UTR genes belonging to all the three different categories in species other than human, are enriched in various other pathways including some disease-related ones. Notably, the short 3′ UTR genes of mouse were found to have the highest number of significantly enriched pathways compared to all other species (Fig. 2).

### Distribution of miRNA binding sites on CNV and non-CNV genes

In order to further examine the differential expression regulation of CNV genes in the different species, we explored the distribution of miRNA binding sites on different regions of the CNV and non-CNV genes. Two prediction algorithms (miRWalk and TargetScan) were employed to generate the possible binding site interactions between human miRNAs and all known genes of 8 different species. The miRNA-target interactions resulting from both algorithms were used to calculate the mean of binding sites per gene for further analysis.

**Figure 3:**
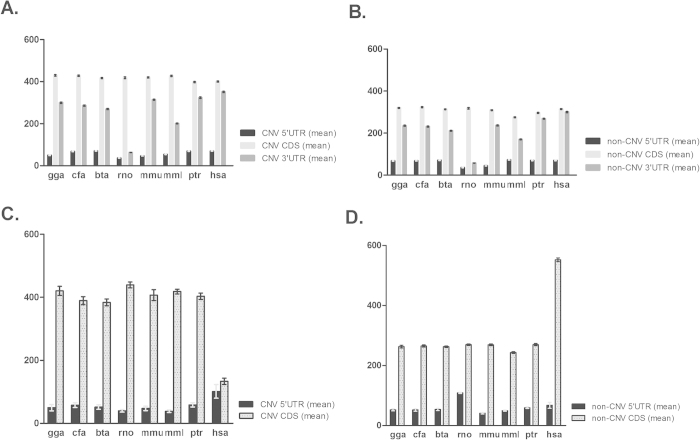
Mean miRNA binding sites per gene in genes with long 3′ UTR (**A**,**B**) and genes with short 3′ UTR (**C**,**D**). A: Mean miRNA binding site/gene on long CNV genes. B: Mean miRNA binding site/gene on long non-CNV genes. C: Mean miRNA binding site/gene on short CNV genes. D: Mean miRNA binding site/gene on short non-CNV genes in the 8 different species (chicken (gga), dog (cfa), cow (bta), rat (rno), mouse (mmu), macaque (mml), chimpanzee (ptr), and human (hsa)). Data represent mean +/− standard error of the mean (SEM).

As expected, human CNV and non-CNV genes with long 3′ UTR were found to have significantly higher number of miRNA binding sites per gene (p < 0.0001; Wilcoxon rank-sum test) on their 3′ UTR regions compared to all the other seven species (Fig. 3A,B and Supplementary Table S1). The miRNA binding sites on other gene regions (5′ UTR and CDS) were not significantly different between human and the other species (Fig. 3A,B, and Supplementary Table S1). On the contrary, in the absence of 3′ UTR (short 3′ UTR genes) human CNV genes were observed to accumulate the higher number of miRNA binding sites per gene (p < 0.0006; Wilcoxon rank-sum test) on their 5′ UTR regions as compared to other species. However, in non-CNV genes, the number of miRNA binding sites per gene on the 5′ UTR was not significantly different (Fig. 3C,D and Supplementary Table S2). Comparisons of the mean length of 5′ UTR, CDS and 3′ UTR between the eight species did not demonstrate any statistical significant variation. As a result, the observed differences in the number of miRNA binding sites cannot be attributed to differences in length of the gene regions (Supplementary Data, Table S3). These results suggest that in the absence of 3′ UTR, CNV genes in humans may specifically accumulate more miRNA binding sites at the 5′ UTR region of mRNA to compensate for the loss of the “canonical” miRNA regulation.

**Figure 4:**
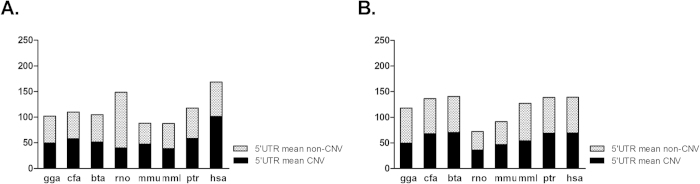
miRNA binding site enrichment analysis in 5′ UTR of CNV versus non-CNV genes having short (**A**) and long 3′ UTRs (**B**). A: Mean miRNA binding sites on the 5′ UTR of CNV versus non-CNV genes with short 3′ UTR among the 8 different species (chicken (gga), dog (cfa), cow (bta), rat (rno), mouse (mmu), macaque (mml), chimpanzee (ptr), and human (hsa)). B: Mean miRNA binding sites on the 5′ UTR of CNV versus non-CNV genes with long 3′ UTR among the 8 different species.

A comparison of the mean miRNA binding sites on the 5′ UTR of long and short 3′ UTR genes between CNV and non-CNV categories demonstrates that although in the long 3′ UTR genes (Fig. 4B) the mean miRNA binding sites are equal or less in CNV genes in all the species examined, in short genes (Fig. 4A) the trend is quite different. The mean miRNA binding sites is significantly higher in human CNV genes (p value = 0.009; Fisher’s exact test) as compared to the CNV genes from the other seven species, highlighting the importance of a tighter expression regulation of human CNV genes expression (Fig. 4).

### Comparison of SNP distribution within 5′ UTR, CDS, 3′ UTR of CNV genes (long 3′ UTR) among different species

SNPs are the most frequent form of variation in genomes. In the human genome specifically more than 38 million SNPs have been identified as part of the 1000 genomes project in 14 different populations^15^. It is well established that this type of variation can affect gene expression. The effects of SNPs on gene expression can occur in two ways changes in the transcript sequences and changes to the regulatory domain (reviewed in^16^). SNPs found in the coding sequence of genes can alter transcript levels by the introduction of premature stop codon and by altering the initial methionine leading to an untranslatable transcript. Similarly, SNPs located on the 3′ UTR can impact miRNA-dependent gene expression regulation by abolishing or creating miRNA binding sites. SNP accumulation would, therefore, be expected to contribute to the genomic and phenotypic variability between species. In an attempt to identify their contribution in expression plasticity we investigated the SNP in both CNV and non-CNV genes in different species.

**Figure 5:**
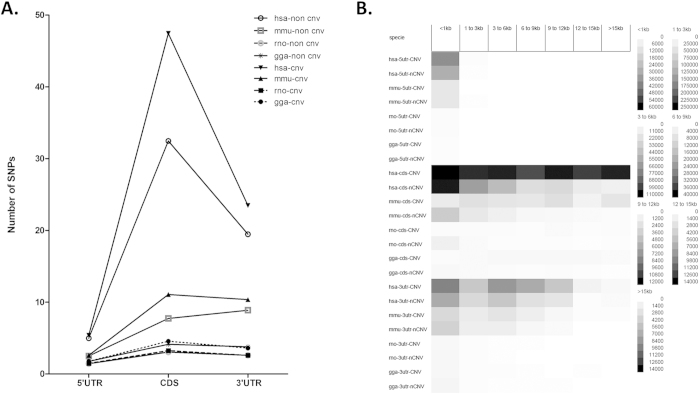
Comparison of mean SNP number and SNP density between CNV and non-CNV genes among human (hsa), mouse (mmu), rat (rno) and chicken (gga). A: Mean number of SNPs distribution between CNV and non-CNV genes in the four different species. B: SNP density cell plot between CNV and non-CNV genes in the four different species.

A comparison of the SNP distribution in four species for which data are available (human, mouse, rat and chicken), indicated that human genes (CNVs and non-CNVs) had significantly higher numbers of SNPs per gene than the other three species (76.30, 24.04, 7.32, and 9.92 in CNV genes and 56.90, 19.12, 7.09, and 9.67 for non-CNV genes, respectively). Similar findings were observed for all gene regions (5′ UTR, CDS and 3′ UTR) with a pronounced difference in the CDS and 3′ UTR (Fig. 5A). The latter is of considerable interest as it signposts a potential interconnection between SNP accumulation and the formation of new miRNA binding sites on human genes. In support to this notion, human CNV genes had a significantly higher number of accumulated SNPs in all gene regions as compared to their non-CNV counterparts (Fig. 5A). In order to avoid any bias due to the differences in 5′ UTR, CDS and 3′ UTR lengths between different species, we calculated the SNP density (SNPs/kb) for each transcript size category (Fig. 5B). In agreement with the previous data, SNP density is higher in humans than in the other three species analyzed for all different gene regions and transcript size categories in support of an increase of miRNA binding sites in human CNV genes. Interestingly, there appears to be an evolutionary trend for the accumulation of SNPs in 3′ UTR of CNV genes, since the number of SNPs in mice is higher in CDS and 3′UTR as compared to rat and chicken that have more SNPs only in CDS regions. When genes with short 3′ UTR were examined, there was no significant redistribution of SNPs on CNV genes, suggesting that a possible SNP-miRNA connection in gene expression plasticity may be confined to the “canonical” miRNA regulation (Supplementary Table S4).

### Differential regulation of CNV and non-CNV genes by “CNV-miRNAs”

**Figure 6:**
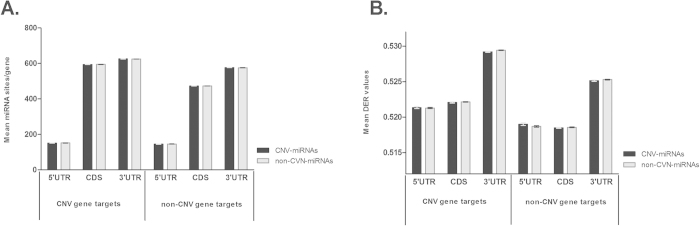
Mean number of miRNA binding sites per gene of CNV and non-CNV miRNAs on human genes (CNVs and non-CNVs). (**A**) Mean value of target genes predicted for CNV- and non-CNV-miRNAs and (**B**). Mean value of DERs of putative targets (human CNV and non-CNV genes) targeted by CNV- and non-CNV miRNAs. Data represent mean +/− standard error of the mean (SEM).

MicroRNAs are major regulators of gene expression and thus appear to play a significant role in phenotypic variability and evolution. A specific subset of miRNA genes localized on CNV regions (CNV-miRNAs) are expected to be pivotal in such processes. CNV-miRNAs were previously shown to be involved in many biological processes and diseases, including organ development, angiogenesis and fertility. Recent studies attempted to map CNV-miRNAs in different human population and unraveled the different layers of genotypic complexities and phenotypic expressions of these molecules^17^. In an attempt to investigate the role of this miRNA subset in human gene regulation, first, a comprehensive atlas of CNV- and non-CNV-miRNAs was constructed by mapping the genomic location of all known human miRNAs against all known human genes. A total of 610 (24%) out of 2,578 human mature miRNAs were located within the CNV regions. These were designated as “CNV-miRNAs”, whereas as the remaining 76% were named “non-CNV-miRNAs”. Secondly, we added the possible interactions of these two groups (CNV-miRNAs and non-CNV-miRNAs) with the genes belonging to CNV and non-CNV regions and compared the mean values of these two miRNA classes within 5′ UTR, CDS and 3′ UTR. As seen in Fig. 6A, the mean value of CNV-miRNAs targeting CNV genes is significantly higher (p < 0.0001; Wilcoxon rank-sum test) compared to the mean values of CNV -miRNAs targeting non-CNV genes in all gene regions (compare black bars in left versus right panel). These results suggest that the regulation of CNV-miRNAs is highly associated with CNV genes. Interestingly, the mean value of non-CNV-miRNAs was also significantly higher (p < 0.0001; Wilcoxon rank-sum test) within CNV genes than the mean value of CNV-miRNAs targeting both categories (CNV as well as non-CNV genes) and non-CNV-miRNAs targeting non-CNV genes. These results also indicate that CNV genes are highly regulated by both classes of miRNAs which further supports the unique and complex regulation of human CNV genes. Comparisons of the mean length values of of 5′ UTR, CDS and 3′ UTR between CNV and non-CNV genes of humans showed no significantly difference (p > 0.05; using ANOVA test) in support of our previous conclusion that the differences were not due to differences in the length of the CNV and non-CNV gene regions (Supplementary data, Table S5).

To further support the notion that the CNV genes are more likely to be regulated by both classes of miRNAs in humans, we downloaded their DERs from the fitSNPs. This DER value represents the prevalence of differential expression of a gene in multiple microarray profiles between thousands of specimens and is estimated by analyzing all the documented human microarray datasets under GEO repository at NCBI. It can be used, therefore, as a comprehensive atlas for the differential expression patterns of human genes at transcriptomic level. It is expected that genes with high DER values will exhibit a more complex and tighter expression regulation. In addition, it has previously been demonstrated that genes with high DER values are more likely to have disease-associated variants^18^. Comparison of the mean values of DERs for the putative targets predicted for CNV- and non-CNV-miRNAs showed that both classes of miRNAs have a significantly higher mean DERs values (p = 0.008 and p = 0.0074 for CNV-, and non-CNV-miRNAs, respectively; Wilcoxon rank-sum test) for CNV genes. These findings further support our previous studies in which we demonstrated that the number of distinct miRNA types and the average number of miRNA binding sites in genes belonging to CNV regions are significantly higher than the ones in non -CNV regions (Fig. 6B).

## Discussion

There is a well-established relationship between changes in gene expression and phenotypic variability at the organismal as well as at the cellular level^19^,^20^. Consequently, by understanding and elucidating the complex networks involved in gene expression regulation will aid in figuring out the phenotypic differences among species under normal and possibly pathological conditions. At the genomic level, various elements interact with each other resulting in a fine-tuned regulation of protein coding genes. Major players in this complex interplay are miRNAs. These short non-coding RNA moieties are well established regulators of gene expression and are involved in a plethora of biological processes. We and others have demonstrated previously that miRNAs have greater potential for interacting with genes found in CNV regions of the genome. In this way, they act as buffers in response to the higher and probable toxic expression of CNV genes during evolution. At the same time, it appears that the interconnection between these two elements follows a unique pattern of regulatory mechanism in the human lineage. As gene expression and regulation can determine to a great degree the phenotype of an organism, the interactions of CNV genes with various genomic elements like miRNAs might help identify unique physiologic properties of humans. In this study, we performed a comprehensive analysis of the regulatory interactions of CNV and non-CNV genes in eight different species (gga, cfa, bta, rno, mmu, mml, ptr, hsa). By using genes with 3′ UTR shorter than 7 nts that lose the “canonical” miRNA-dependent regulation as a model we were able to explore the nature of CNV genes expression regulation and examine the differences between human and other species.

Humans appear to have significantly fewer genes with short 3′ UTRs than all the other seven species investigated. The small number of such genes highlights the importance of miRNA-dependent regulation in the human lineage. This observation together with our previous data, in that human genes are regulate by more miRNAs than their homologues in other species, indicate that human genes are under a tighter regulation regiment.

Surprisingly, though, when we divided those genes into CNV and non-CNV categories, we observed that while in all the other species the majority of short 3′ UTR genes belongs in the non-CNV category, human follow the exact opposite trend suggesting an alternative mechanism of regulation of these genes in human lineage. If CNV genes require a higher degree of miRNA regulation, then why are these short 3′ UTR genes enriched in that category? Assuming that the formation of new miRNA binding sites on genes is linked directly or indirectly to CNV-formation, short 3′ UTR genes in humans will be pertained with a higher frequency in CNV regions, as these genes may favor the formation of miRNA binding sites in the regions such as the 5′ UTR. Indeed, our data demonstrated that in the absence of 3′ UTR, the category of CNV genes in humans specifically accumulate more miRNA binding sites on the mRNA 5′ UTR region. This may represent a compensatory mechanism in response to the loss of the “canonical” miRNA regulation. Evolution of the “non-canonical” regulation will probably be favored only on CNV regions in the human lineage.

Pathway analysis of the short 3′ UTR genes revealed two pathways which are significantly enriched in the majority of species in all the three categories. The Olfactory- and Taste-transduction pathways were found significant for six (cfa, hsa, mml, mmu, ptr and rno) out of eight species. It is interesting to note that in humans, genes with short 3′ UTRs are enriched only in the above mentioned pathways. As these genes lose the “canonical” miRNA-dependent regulation, they are expected to have deregulated levels of expression and mRNA stability^21^,^22^. However, the presence of such genes might not be permitted in dosage-sensitive or disease-related pathways in humans. In support of this, alternative polyadenylation and 3′ UTR shortening were observed in carcinogenesis^22^. In addition, two species, cow and mouse, seem to be of considerable interest with regard to short 3′ UTR gene functional distribution. None of the identified pathways in this study were significant in cow. On the contrary, most of the identified pathways were found significant in mouse. Although, these observations cannot be readily explained from our data, the enrichment of many disease-related pathways with genes with reduced expression regulation, might question the suitability of the use of mouse as a model for various diseases. On the other hand, our results suggest that the rat profile may resemble more the human profile. This difference in expression regulation between the species may obscure scientific conclusions or result in false responses. Therefore, expression profiling studies between mouse and human in response to pathological interventions would be required to further support this hypothesis.

So what is happening when genes loose the “canonical” miRNA regulation? Numerous studies have demonstrated that miRNAs can bind in a “non-canonical” fashion on the 5′ UTR and coding sequences of mRNAs and regulate their expression^23^,^24^,^25^,^26^ in humans and drosophila. So far, only a few resources such as miRWalk^27^ supply miRNA binding site predictions within the complete sequence (promoter, CDS, 5′ and 3′UTR regions) of genes. Such binding interactions can result in both translational activation and translational inhibition via different mechanisms. The increased number of miRNA binding sites observed on the 5′ UTR of human CNV genes lacking 3′ UTR (Fig. 3), may indicate the presence of an evolutionary compensatory mechanism to counteract the absence of “canonical” miRNA-mediated expression regulation for those genes. This appears to be human specific reinforcing the notion that human CNV genes are under a tighter gene expression regulation than in other species. A similar pattern was observed during the SNP distribution analysis on CNV and non-CNV genes. Moreover, all human genes demonstrated a higher SNP density in all gene regions as compared with other species indicating an evolutionary connection with miRNA binding site accumulation that is confined to the “canonical” miRNA-dependent regulation.

The complex and rigid expression regulation of CNV genes is also evident from their higher DER values. Is this tight regulation advantageous for the human lineage? Certainly, more studies are required to confirm this. One could assume that this differential CNV gene regulation may have contributed to the specific physiologic characteristics of the human species. At the same time, however, it may act as an obstacle to any spontaneous compensation of gene expression levels under a pathologic insult and contribute to the development and progression of human diseases.

It should be emphasized that the results of this study should be interpreted with caution. Compared to humans, genomic data in other species are limited. As a result, we had to utilize human miRNAs and human CNV genes to interrogate other species genomes. This might create an unavoidable bias. More importantly, SNP comparison analysis is only indicative of a potential trend. As a result of numerous population studies in humans many more SNPs were discovered as compared with other species. We attempted to minimize the aforementioned limitations by data normalization and distribution analysis but certainly further validation is needed. Nevertheless, despite these limitations our data support the notion of a complex and tight regulation of CNV genes in general and specifically of human CNV genes.

## Materials and Methods

### Compilation of CNV and non-CNV genes, and their classification into long, and short 3′ UTR

Homologous CNV and non-CNV gene information acquisition was accomplished as described by^28^. Briefly, first, the information on CNV and all known human genes was collected from the Database of Genomic Variants^29^ and Gene database ( ftp://ftp.ncbi.nih.gov/gene/DATA/), respectively. Second, the homologous gene datasets for 7 other species: chicken (gga), dog (cfa), cow (bta), rat (rno), mouse (mmu), macaque (mml) and chimpanzee (ptr) were gathered from HomoloGene ( ftp://ftp.ncbi.nih.gov/pub/HomoloGene/). Third, in order to build a comprehensive list of homologous CNV and non-CNV gene categories, human CNV genes were mapped to homologous gene datasets of 7 other species (Supplementary Data as Table S6). Thereafter, these two categories (CNV and non-CNV genes) were further classified into two different classes: long and short 3′ UTR genes, by estimating 3′ UTR length of all known genes of the 8 species. In order to estimate 3′ UTR lengths, information on all known mRNAs of the 8 species was downloaded from RefSeq ( ftp://ftp.ncbi.nih.gov/refseq/)^30^. A customized “Perl script” ( http://www.perl.com) was written to parse only the information of interest (EntrezID, RefseqID, mRNA length, CDS start and end positions). The parsed data (mRNA information) was then compiled in a file and mapped to CNV and non-CNV genes. During this mapping step, some of these genes were found to match with more than one transcript; therefore, in order to maintain this list non-redundant, only the longest mRNAs were selected for every gene. Following this, the 3′ UTR length for each gene was calculated by subtracting the end positon of CDS region from its mRNA length and the genes were categorized into two classes: long and short 3′ UTR. The long 3′ UTR class contains those genes having at least 8nt long 3′ UTRs, whereas the remaining genes were assigned under the short 3′ UTR class.

### Classification of human miRNAs into CNV- and non-CNV classes

The sequence of human miRNAs (n = 2,578) and their coordinates were obtained from miRBase release 20 ( ftp://mirbase.org/pub/mirbase/20/)^31^, whereas, the genomic coordinates of human CNV genes were downloaded from NCBI ftp site ( ftp://ftp.ncbi.nlm.nih.gov/gene/DATA/). Following this, the genomic locations of downloaded miRNAs were scanned against the coordinates of human CNV genes by a customized “Perl script”. The miRNAs that overlapped within CNV regions were designated as “CNV-miRNAs”, while, the remaining miRNAs were termed as “non-CNV-miRNAs”.

### miRNA binding site predictions and their enrichment analysis within CDS, 5′ and 3′ UTR regions

The mRNA sequences of CNV and non-CNV genes of 8 species were retrieved from RefSeq ( ftp://ftp.ncbi.nih.gov/refseq/). A screening was carried out by locally executing two different miRNA-target prediction algorithms: miRWalk ( http://zmf.umm.uni-heidelberg.de/apps/zmf/mirwalk2/)^27^ and Targetscan^32^ to identify the possible interactions between human miRNAs and the downloaded sequences of CNV, and non-CNV genes of 8 species. It has previously been shown that by considering the miRNA binding site prediction datasets resulting from different algorithms helps to minimize both, the number of putative target genes as well as false positive targets genes^7^,^28^,^33^. Information on miRNA-target interactions obtained from the miRWalk algorithm (16 datasets: CNV and non-CNV) and Targetscan (16 datasets: CNV and non-CNV) was preprocessed to remove unnecessary data and separated into several lists (n = 32). To attain only those interactions predicted with both algorithms, the lists of Targetscan were then mapped to the ones resulted from miRWalk as described elsewhere^7^,^28^. Thereafter, the binding site interactions between human miRNAs and CNV and non-CNV genes predicted with both algorithms were compiled and subjected to further analysis. To identify the enrichment of miRNA binding site within CNV and non-CNV categories including short and long 3′ UTR genes, a customized “R script” was employed using Fisher’s exact test with Benjamini and Holm (BH) method with 5% level of significance as previously described 7. Briefly, first, the total number of miRNA binding sites within 5′ UTR, CDS and 3′ UTR regions of all known genes (CNV and non-CNV) of 8 species was computed. These values were further stored into various files (for example, cnv-ncnv.txt i.e., comparison between CNV and non-CNV genes including short and long 3′ UTRs) having 5 columns. In a next step, an overrepresentation analysis was carried out to evaluate the enrichment of miRNA binding sites within the different regions of known genes belonging to short and/or long 3′ UTR (CNV and non-CNV categories).

### Pathway enrichment analysis

The short 3′ UTR homologous genes belonging to two different categories (CNV and non-CNV regions) of 8 species were interrogated to identify significantly enriched KEGG pathways^34^ using a customized “R script” by implementing Fisher’s exact test with the Benjamini and Hochberg (BH) as a multiple testing (background correction) method with 5% level of significance.

### Acquisition of DERs and SNPs

Differential Expression Ration (DERs) values for human genes were attained from fitSNPs database^18^. The DER value of a human gene represents its observed frequency of differential expression that is calculated by analyzing all human microarray profiling studies documented under the Gene Expression Omnibus (GEO at http://www.ncbi.nlm.nih.gov/geo/).

Information on single nucleotide polymorphisms (SNPs) on CDS, 5′ and 3′ UTR regions of human, mouse, rat and chicken was extracted from SNP database by using the user interface ( http://www.ncbi.nlm.nih.gov/snp/limits).

### Statistical comparison of mean values

In order to evaluate the statistical significance on the mean values (such as miRNA binding sites, SNPs and DERs per gene) among different species, analysis of variance (ANOVA) was firstly applied to test the null hypothesis (i.e., H_o_: all means are equal) followed by the multiple comparison using Wilcoxon rank-sum test with 5% level of significance (p < 0.05).

## Additional Information

**How to cite this article**: Dweep, H. *et al.* Homo sapiens exhibit a distinct pattern of CNV genes regulation: an important role of miRNAs and SNPs in expression plasticity. *Sci. Rep.* **5**, 12163; doi: 10.1038/srep12163 (2015).

## Supplementary Material

Supplementary Information
