# Supplementary material for: Homo sapiens exhibit a distinct pattern of CNV genes regulation: an important role of miRNAs and SNPs in expression plasticity
Source: Sci Rep. 2015 Jul 16;5:12163. doi: 10.1038/srep12163 (PMC4503977; doi:10.1038/srep12163)
Supplement: Supplementary Information [file srep12163-s1.doc]

**Supplementary Information**

**Homo sapiens exhibit a distinct pattern of CNV genes regulation: an important role of miRNAs and SNPs in expression plasticity.**

Harsh Dweep^1^, Nada Kubikova^2^, Norbert Gretz^1^, Konstantinos Voskarides^3^, Kyriacos Felekkis^2,4*^

**Table S1:** Mean miRNAs binding sites within 5' UTR and CDS regions of CNV and non CNV genes (short 3' UTR) among 8 species.

|  | **CNV** | | **nonCNV** | |
| --- | --- | --- | --- | --- |
| **Region** | **5utr (mean)** | **Cds (mean)** | **5utr (mean)** | **Cds (mean)** |
| **bta** | 51.61 | 383.7 | 53.33 | 262.4 |
| **cfa** | 57.94 | 389.46 | 51.96 | 264.72 |
| **gga** | 49.7 | 420.54 | 52.26 | 262.77 |
| **hsa** | 101.27 | 133.57 | 67.14 | 552.17 |
| **mml** | 38.63 | 418.35 | 49.27 | 242.98 |
| **mmu** | 47.46 | 406.92 | 40.7 | 268.89 |
| **ptr** | 58.58 | 403.3 | 59.21 | 269.52 |
| **rno** | 39.93 | 439.31 | 108.47 | 269.4 |

**Table S2:** Mean miRNAs binding sites within 5' and 3' UTR, and coding sequence (CDS) regions of CNV and non CNV genes (long 3’-UTR) among 8 species.

|  | **CNV** | | | **Non-CNV** | | |
| --- | --- | --- | --- | --- | --- | --- |
| **Region** | **5utr (mean)** | **Cds (mean)** | **3utr (mean)** | **5utr (mean)** | **Cds (mean)** | **3utr (mean)** |
| **bta** | 70.2354024 | 416.824288 | 269.308931 | 70.5933439 | 313.330046 | 210.851892 |
| **cfa** | 67.8883851 | 428.396145 | 286.17747 | 68.3446843 | 323.326179 | 231.309768 |
| **gga** | 49.5771628 | 429.874444 | 299.790092 | 68.394438 | 320.068821 | 234.877863 |
| **hsa** | 69.3941783 | 400.581584 | 351.359836 | 69.6696576 | 314.339318 | 300.324458 |
| **mml** | 53.9866002 | 427.619708 | 200.962177 | 72.9163239 | 275.169701 | 169.889939 |
| **mmu** | 46.9754303 | 420.093584 | 314.41225 | 44.4650993 | 309.10762 | 236.423054 |
| **ptr** | 69.0631715 | 397.884911 | 323.926787 | 69.5533615 | 296.501306 | 268.229996 |
| **rno** | 36.2897503 | 418.631946 | 62.829364 | 35.9658186 | 318.218503 | 57.4093137 |

**Table S3: Comparison of the mean length values of 5' UTR, CDS and 3' UTR between the eight different species plus Standard Error of the Mean (SEM).**

| **Species** | **5UTR (Mean)** | **5UTR (SEM)** | **CDS (Mean)** | **CDS (SEM)** | **3UTR (Mean)** | **3UTR (SEM)** |
| --- | --- | --- | --- | --- | --- | --- |
| **bta** | 242.125779 | 2.73394731 | 1581.33092 | 11.0056606 | 852.459186 | 7.23909051 |
| **cfa** | 240.078348 | 2.85455363 | 1695.66920 | 12.5537973 | 980.079605 | 8.85631590 |
| **gga** | 215.734807 | 3.76270854 | 1696.14417 | 13.6650300 | 1070.60224 | 11.1628468 |
| **hsa** | 290.044201 | 2.47438123 | 1704.99456 | 12.9459341 | 1399.41699 | 11.2033989 |
| **mml** | 182.213078 | 2.03779757 | 1450.33410 | 10.8577348 | 578.078972 | 5.58760685 |
| **mmu** | 174.111191 | 1.35600080 | 1596.92187 | 11.1346280 | 1023.16805 | 8.48748668 |
| **ptr** | 234.506028 | 2.08042561 | 1534.57258 | 10.1989425 | 1094.85799 | 9.21800925 |

**Table S4**: Comparison of number of SNPs within 5' UTR, CDS and 3' UTR of CNV genes (both short and long 3' UTRs) versus non CNV genes (both) among hsa, mmu, rno, gga, mml, ptr using Fisher’s exact test with BH as multiple testing method with 5% level of significance.

| **Comparison** | **SNPs in CNV** | **CNV genes** | **SNPs in non CNV** | **Non CNV genes** | **p-value** | **p-value BH** | **Fold enrichment** |
| --- | --- | --- | --- | --- | --- | --- | --- |
| **hsa-cds-both-shorter-longer** | 478520 | 10084 | 268021 | 8254 | 2.78E-138 | 3.89E-137 | 1.461316325 |
| **mmu-cds-both-shorter-longer** | 91301 | 8245 | 73593 | 9504 | 1.98E-113 | 1.38E-112 | 1.430039677 |
| **hsa-3utr-both-shorter-longer** | 223364 | 9517 | 146504 | 7528 | 1.72E-32 | 8.03E-32 | 1.205979256 |
| **mmu-3utr-both-shorter-longer** | 76601 | 7384 | 63742 | 7177 | 2.75E-19 | 9.62E-19 | 1.168042218 |
| **hsa-5utr-both-shorter-longer** | 42938 | 7984 | 32162 | 6463 | 1.16E-05 | 3.24E-05 | 1.08071644 |
| **gga-cds-both-shorter-longer** | 19767 | 4331 | 16146 | 3908 | 2.53E-05 | 5.91E-05 | 1.104690941 |
| **rno-cds-both-shorter-longer** | 12897 | 3963 | 17030 | 5582 | 0.003467238 | 0.006934477 | 1.066720348 |
| **mmu-5utr-both-shorter-longer** | 10570 | 4080 | 10700 | 4276 | 0.091662794 | 0.160409889 | 1.035318339 |
| **rno-5utr-both-shorter-longer** | 1480 | 1001 | 1474 | 1025 | 0.325556315 | 0.506420934 | 1.028149017 |
| **gga-5utr-both-shorter-longer** | 783 | 447 | 889 | 506 | 0.530933042 | 0.743306259 | 0.997018993 |
| **rno-3utr-both-shorter-longer** | 7146 | 2762 | 7411 | 2846 | 0.587464489 | 0.747682077 | 0.99356741 |
| **ptr-cds-both-shorter-longer** | 2311 | 1287 | 2356 | 1242 | 0.87198547 | 1 | 0.946637098 |
| **gga-3utr-both-shorter-longer** | 9764 | 2707 | 9400 | 2484 | 0.939170026 | 1 | 0.953136291 |
| **mml-cds-both-shorter-longer** | 9136 | 2884 | 17258 | 3730 | 1 | 1 | 0.684704052 |

**Table S5:** Comparison of the mean length values of 5' UTR, CDS and 3' UTR between human CNV and non-CNV genes plus Standard Error of the Mean (SEM).

| **Region** | **Mean** | **SEM** |
| --- | --- | --- |
| **CNV-3UTR** | 1585.5 | 16.71 |
| **CNV-5UTR** | 315.71 | 3.5 |
| **CNV-CDS** | 1958 | 21.19 |
| **non-CNV-3UTR** | 1319.28 | 15.4 |
| **non-CNV-5UTR** | 301.92 | 3.79 |
| **non-CNV-CDS** | 1424.93 | 13.33 |

**Table S6**: Total number of CNV and non-CNV genes in the 8 different species.

| **Species** | **CNV (N)** | **Non-CNV (N)** |
| --- | --- | --- |
| hsa | 10,832 | 9,190 |
| mmu | 9,513 | 12,719 |
| ptr | 9,324 | 12,784 |
| cfa | 9,051 | 10,821 |
| bta | 8,996 | 13,925 |
| mml | 8,633 | 13,894 |
| gga | 7,077 | 10,069 |
| rno | 4,629 | 10,043 |
